# Experimental Researches Applied to Physiology and Pathology

**Published:** 1853-05

**Authors:** E. Brown-Séquard

**Affiliations:** Paris


### *Experimental Researches applied to Physiology and Pathology.*

By E. Brown-Séquard, M. D., of Paris.

(Continued.)

### XXVIII.—INFLUENCE OF RED BLOOD ON MUSCLES AND NERVES DEPRIVED OF THEIR VITAL PROPERTIES.

James Phillips Kay* has found that blood, injected into limbs of dead animals, just after irritability has disappeared, is capable of regenerating this vital property. I have gone much farther, and have discovered that blood is able to regenerate the vital properties of nerves and muscles, even in limbs which have lost their irritability and have been rigid for several hours. I have obtained this result from the following experiments:

1st. On the body of a rabbit, in which cadaveric rigidity had already existed for 10, 20, and in one case, 33 minutes, I divided the aorta and the vena cava in the abdomen, immediately

* Treatise on Asphyxia. London, 1834

above the bifurcation of these vessels. By means of small tubes, a communication was established between their peripheric extremity and the central extremity of the corresponding vessels divided in a living rabbit. The blood of this living animal circulated immediately in the posterior limbs of the dead one. After about six, eight or ten minutes, rigidity disappeared, and, a few minutes afterwards, movements took place when I excited the muscles or the muscular nerves.

2d. I have obtained a like result from an experiment more easily made than the preceding, and which I have performed more frequently. I divided transversely the body of a living guinea-pig, or rabbit, into two halves, on a level with the lower border of the kidneys, leaving no communication between the two halves, except by the aorta and the vena cava. I then tied the aorta immediately below the origin of the renal arteries. The muscular irritability gradually diminished, and in a very variable length of time* it gave way to cadaveric rigidity. I waited until rigidity had been fully developed in all the muscles, and then the ligature was relaxed and the circulation re-established. Rigidity disappeared slowly, and the muscles and the motor nerves resumed their vital properties.

3d. In order to ascertain if voluntary movements and sensibility could be restored to limbs that had been in a state of cadaveric rigidity, I tied the aorta immediately behind the origin of the renal arteries, in several rabbits. Shortly afterwards, sensibility and the voluntary movements disappeared in the posterior limbs. I waited until muscular irritability had given way to what is called *cadaveric* rigidity; and when that peculiar rigidity had existed for at least twenty minutes, I relaxed th ligature. Then circulation took place, and, in consequence of it, sensibility and voluntary movements re-appeared.

From this experiment it results, that not only local life, but all the properties and actions of full life, can be restored in limbs that have been in the state called *rigor mortis, cadaveric or post-mortem rigidity*.

4th. On a man, 20 years old, who was guillotined on the

* Sometimes 30, 20, or even only 10 minutes in weak animals, and from 1 to 8 or 9 hours in strong animals.

18th of June, 1851, in Paris, I made an experiment similar to some of the preceding. The decapitation took place at 8 o'clock A. M. Ten hours afterwards, *i. e.* ten minutes after 6 o'clock P. M., the muscles of the hand, upon which I intended to experiment, exhibited some slight manifestations of irritability. At 7 and at 7½ o'clock P. M. I ascertained that they had lost their irritability. Shortly after they were in a state of cadaveric rigidity.

I began the injection of blood 10 minutes after 9 o'clock P. M.

As I wished to inject fresh human blood, and as I could not obtain any from the hospitals at such an hour, I was obliged to make use of my own. My friends, Drs. F. Bonnefin and Deslauriers, drew from one of the veins of my left arm half a pound of blood, which was immediately beaten and completely defibrinated and filtered through a cloth.

As, in opposition to the general opinion, I had found that it is not necessary, in transfusion, to make use of blood at a temperature not far from that of warm-blood animals, I left the blood employed in this experiment freely exposed to the atmosphere during all the time of the operation. The temperature of the air was 19° centigr. (66°·2 Fahr.) I regret not having taken the temperature of the blood when I began to inject it, but it was probably about the same as that of the atmosphere.

The injection was made into the radial artery, a little above the wrist. The whole quantity of the blood was injected in about 8 or 10 minutes. The arm operated on had been separated from the body, and the blood injected came out from all the divided arteries and veins.

Having saved nearly all the blood which flowed from these vessels, I injected it anew. The last injection was made 45 minutes after 9 o'clock P. M. Ten minutes afterwards I found that cadaveric rigidity had ceased in the hand, and that two muscles only, out of the nineteen existing in that part, had not resumed their irritability. Three muscles had become so very irritable that a slight mechanical excitation was followed by a contraction in the whole length of their fibres.

At half past one o'clock A. M.,—seventeen hours and a half after decapitation and four hours after the injection of blood,—there was still a slight irritability in the muscles of the hand.

In this experiment I found that half a pound of defibrinated human blood was sufficient to give irritability, for several hours, to seventeen of the muscles of a hand.*

5th. An experiment on another guillotined man gave me more interesting results. The decapitation had taken place at 8 o'clock A. M. on the 12th of July, 1851. At 5½ o'clock P. M., cadaveric rigidity existed in almost all the muscles of the arms and fore-arms. I separated them from the body, and at 6½ o'clock I ascertained that cadaveric rigidity was increased, and that only a few muscles were still slightly irritable. At 8 o'clock P. M. (12 hours after the decapitation) the muscles of the two arms were completely deprived of irritability, and in full rigidity, and the muscles of the forearms contracted only locally under the influence of a mechanical irritation, and not at all when excited by a powerful magneto-electric current. Two other examinations made, one at 9½ and the other at 10 o'clock, gave the same results.

At 10¼ o'clock two or three bundles of fibres of one of the muscles of the fore-arm were the only parts where a mechanical excitation produced a slight local contraction. All the other muscles were perfectly stiff and deprived of irritability.

Twenty-five minutes after 10 o'clock there was no appearance of irritability remaining in any muscle.

I then began the preparations for the injection of blood, with the assistance of Drs. Martin-Magron, F. Bonnefin, Crouzet, and Mr. Moyse.

We drew about a pound of blood from the carotid of a strong dog. The blood was beaten and defibrinated before coagulation could take place in it, and 10 minutes after 11 o'clock the injection was begun. It was made in the brachial artery of the left arm, in the middle of its length, where the arm had been amputated. As soon as the blood had been thrown in the artery, some reddish spots appeared in different parts of the skin of the fore-arm, of the hand, and more particularly of the wrist. These spots became larger and larger, and the skin had the appearance it has in rubeola. Soon after, the whole surface of the skin was of a violet reddish hue. In a few minutes this color disappeared,

* For a full account of the circumstances of this experiment, see my paper in the *Gaz. Medic. de Paris*, t. vi.—1851, p. 421.

and was replaced by the natural hue of the skin during life. The skin became elastic and soft, as in a living man, and we saw the bulbs of its hair becoming erected and presenting the appearance called *cutis anserina.* By increasing and diminishing alternately the impulsion given to the blood, we succeeded in producing the beatings of the pulse in the radial artery. The veins were distinct and full as during life.

A short time after, the fingers, which had been extremely stiff, relaxed, and rigidity disappeared also in the other parts of the limb.

Forty-five minutes after 11 o'clock P. M., irritability had returned in all the muscles of the limb operated on. The degree of irritability, more particularly in the muscles of the arm, (triceps, biceps and others) was very considerable, and much greater than I had seen it at the time the corpse was first examined (about five o'clock P. M.) Irritability was still present in almost all the muscles of this limb at 4 o'clock A. M., (20 hours after the decapitation,) when I was obliged, from extreme fatigue, to abandon further investigation.

The blood injected was at 23° centig. (73°.4 Fahr.) when I began the operation, and the atmosphere was at $19\frac{1}{4}$° centig. (66°.66 Fahr.)

In this experiment, about one pound of defibrinated dog's blood gave irritability for more than five hours to all the muscles of a limb, from the middle of the arm to the hand.

6th. Every one knows the singular fact, that Vibriones and other Infusoria, when desiccated, will live when they are put into water. It is also perfectly known that seeds, after many centuries, may grow when put in the earth. I have found something of the same kind in higher organisms; it is that muscles, in a certain condition, after having been separated from the body for many days, may recover their irritability.

Dr. Coze, of Strasbourg, has found that chloroform injected into the main artery of a limb produces instantly the strongest rigidity, and that if blood is allowed to circulate again in the limb, life appears again in it. I have gone farther, and found that if a limb, in which an injection of chloroform has been made, is separated from the body, it is able, under the influence of an injection of blood, to recover its muscular irritability 2, 3, 4, 5

and (in one case) 10 days after the rigidity was produced. I think Mr. Edouard Robin is right in admitting that chloroform prevents the chemical changes that take place in organic bodies after death, and, if it is so, we can understand why an injection of blood made so long after the limb has been separated from the body, may reproduce irritability. One day is not more than one hour, if, during it, there is no alteration produced in the muscles.

It appears, nevertheless, that chloroform does not entirely prevent the alterations of muscles, because, in my experiments, I have found that the longer the limbs had been separated from the body, the greater was the quantity of blood necessary to reproduce irritability.

7th. I lately made an experiment, with the view of ascertaining how long a limb, separated from the body of an animal, may be kept alive by means of injected blood. I succeeded in retaining local life in one of the limbs of a rabbit more than 41 hours. The animal was a very vigorous, full grown one. I killed it by hemorrhage, and, two hours afterwards, rigidity had begun in most of the muscles of the two posterior limbs, and only a few bundles of muscular fibres had still a slight irritability. A first injection of defibrinated blood was then pushed in the femoral artery of the right posterior limb. Fifteen minutes after the beginning of the injection, local life (*i. e.* irritability) was restored in the limb receiving blood, and cadaveric rigidity had disappeared.

The manner of testing this irritability was the same as that of Glisson, Gorter, and all the experimenters of the two last centuries,—I mean by mechanical excitation. I did not use galvanism, as it exhausts muscular irritability too much, as Autenrieth, Pfaff and many other observers have shown long ago. Being aware of this fact, I have always, in my preceding experiments, made use of galvanism for a very short time only.

Three hours after the death of the rabbit, irritability still existed in the right limb (the injected one,) while the left was perfectly rigid and had not the slightest irritability. Half an hour later, rigidity had begun again in the right limb; blood was injected anew, rigidity disappeared, and local life returned. From this moment until 11 o'clock, P. M., (death had occurred at 6 o'clock, A. M. of the same day,) blood was injected many times. Rigidity did not return, and the vital property of the muscles was maintained. Of course the left limb, during that time, remained rigid, and had not the slightest irritability.

From 11 o'clock, P. M. until 6 o'clock, A. M. the succeeding day, an abundant injection of blood was made every twenty or twenty-five minutes. The irritability was not powerful, but it existed in all the muscles of the limb. There was no rigidity at all.

The injections were then made more frequently—once in each quarter of an hour—until three o'clock, P. M., at which time I was obliged to stop them for an hour and a half.

At half past four I found the limb rigid, and only a few bundles of muscular fibres still irritable. A very abundant injection was then practised, and rigidity soon disappeared, giving way to irritability. From this time to 11 P. M., a great many injections were made, and irritability was maintained. I was then obliged to give up the experiment. At that moment irritability was strong in all the muscles of the injected limb, except some parts of their pelvic extremities that had not received a sufficient quantity of blood.

The next morning that limb was in full and energetic rigidity. The other limb had already lost its rigidity, and had an evident smell of putrefaction. The third day after the death of the animal, rigidity was strong in the injected limb, while the other was in an advanced state of putrefaction.

If we compare these two limbs, we find, 1, That the injected one had a strong irritability at the end of forty-one hours after the death of the animal; 2, That its rigidity gave way to putrefaction only at the eightieth hour; 3, That it was in complete putrefaction only at the ninety-fourth hour. The other limb was in full rigidity at the fifth hour after the death of the animal; its rigidity gave way to putrefaction at the forty-eighth hour; and it was in complete putrefaction at the seventieth hour.

From all the experiments above related, it appears that life may be reproduced or maintained in muscles and nerves by mere injections of blood. I have found, also, that life may be reproduced by the same means in the spinal cord and in the brain. I will publish these facts in another article.

It is nearly indifferent in these experiments whether we use venous or arterial blood; but it is absolutely necessary to employ red blood, *i. e.* oxygenated blood.

I have tried, sometimes, arterial blood, rendered black by the substitution of nitrogen or hydrogen for a great part of its oxygen, and I have found that such blood was unable to reproduce the vital properties of nerves and muscles.

Oxygen is necessary, either because it prevents the blood-globules from being altered, or because it acts directly on muscles, as Gustavus Liebig has found it does on their external surface, when exposed to air. I believe it is necessary for both these reasons.

I cannot say how long after the beginning of cadaveric rigidity in a muscle, oxygenated blood can reproduce local life. In the second of the two decapitated men, on whom I experimented, rigidity had existed at least five hours before the injection was begun. I believe that the stronger the animal is, the more easy it is to reproduce local life in rigid limbs, by injection of blood. In limbs of weak rabbits, I have found it impossible, two hours after the beginning of cadaveric rigidity, to reproduce local life. In a very strong dog I have reproduced muscular irritability four hours after rigidity had been fully developed.

Ten, twelve, or fourteen hours after rigidity had taken place, in human limbs, I have tried in vain to re-establish local life.

I have ascertained that pure serum of blood, or milk, or albumen of eggs, are unable to produce any apparent change in rigid limbs.

The following conclusions are to be drawn from the facts related in this article:

1st. Red blood, *i. e.* richly oxygenated blood (arterial or venous) is able to revive irritability in muscles, four or five hours after these organs have lost this property.

2d. Red blood is able to revive the vital properties of nerves and nervous centres, when these properties have not been lost for more than about an hour.

3d. Muscular irritability can be maintained for more than 41 hours, by mere injections of blood, in limbs separated from the body of a rabbit.

4th. Muscular irritability may be re-established in limbs rendered rigid by chloroform for many days, even ten days.

XXIX.—CASES OF LOSS OF SENSIBILITY ON ONE SIDE OF THE BODY, AND LOSS OF VOLUNTARY MOVEMENTS ON THE OTHER SIDE.

It has been objected to me that if the transmission of sensitive impressions, in the spinal cord, takes place, as I have tried to prove in a former part of this sketch (Art. XIX,) so that those coming from the left side of the body, are mostly conveyed to the sensorium along the right side of the spinal cord,—*et vice versa*—physicians should have some times found in man the same thing that I have discovered in animals.

Many reasons have prevented physicians from making such a discovery: In the first place, an injury or a pathological alteration, limited to a lateral half of the spinal cord, is very rare. Besides, the idea that there is no crossing of fibres in the spinal cord, has been an obstacle to a thorough examination of many pathological cases, and it has been so in a case observed by Boyer.

There are but few cases on record in which there was a loss or a diminution of sensibility on one side, and of voluntary movements on the other. I will give here a short account of some cases of that kind, which are very interesting.

The first one I will relate has been observed by Boyer:

A drummer, of the National Guard of Paris, received a wound in the back of his neck. A sword had been thrown at him, and had penetrated the superior part of the right lateral half of the neck. An incomplete paralysis of movement took place in the right side of the body, and, some time after, it was accidentally discovered that sensibility was lost in many parts of the left side of the body. After twenty days the wound was cured, and the man went out of the hospital, still paralysed.

From what we know of that case, it appears that the sword had incompletely divided the right lateral half of the spinal cord. The paralysis of motion on the right side of the body was certainly produced by the division of a part of the anterior column, and, as the instrument had penetrated the right side of the back of the neck, it must have divided the parts between the anterior column of the spinal marrow and the external surface of the right side of the neck. These parts, besides the muscles and bones, are the lateral and posterior columns and the gray matter of the right half of the spinal cord. So that in this case nearly the same injury and also the same morbid phenomena had existed as in the animals on which I have divided a lateral half of the spinal cord.

The following case is still more interesting. It has been recorded by Dr. R. Dundas, Surgeon of the Hospital of Bahia.

A mason fell on his back from an height of 20 feet. After having recovered his consciousness, he discovered that all the left side of his body, from the shoulder to the foot, was paralyzed as to motion, without the slightest alteration of sensibility, and that the right side in which the movements were free, was completely deprived of sensibility.

Three important facts, precisely like those I have discovered in animals after the transversal section of a lateral half of the spinal cord, existed in this case :

1st. A morbid exaltation of sensibility in the side where movement was lost.

2d. A diminution of temperature in the side where the paralysis of sensibility existed.

3d. An increase in temperature in the side where the paralysis of movement existed.*

When Dr. Dundas published this curious case, the patient was living and improving ; so we do not know what was the alteration existing in the spinal cord.

H. Ley, in a letter to Sir Charles Bell, relates the following case :†

Mrs. W., after a profuse hemorrhage, became paralytic. Upon one side of the body there was a loss of sensibility, without, however, any corresponding diminution of power in the muscles of volition. The breast, too, upon that side, partook of the insen-

* In a former part of this sketch (Art. xxii.) I have related facts proving that animal heat may be increased after injuries to the spinal cord. I have learned since, that Prof. D. Gilbert has observed a case of fracture of the spine, in which the temperature of the paralyzed parts was increased. Prof. Dunglison has also stated that the paralyzed side in hemiplegic patients may have an elevation of temperature.

†The nervous system of the human body. By Ch. Bell. 3d ed. London, 1844, p. 245.

sibility, although the secretion of milk was as copious as in the other. She could see the child sucking and swallowing, but she had no consciousness, from feeling, that the child was so occupied.

Upon the opposite side of the body there was defective power of motion, without, however, any diminution of sensibility. The arm was incapable of supporting the child; the hand was powerless in its grasp; and the leg was moved with difficulty, and with the ordinary rotatory movement of a paralytic patient; but the power of sensation was so far from being impaired that she constantly complained of an uncomfortable sense of heat, a painful tingling, and more than the usual degree of uneasiness from pressure, or other modes of slight mechanical violence.

She again proved pregnant. Her delivery was easy: but after about ten days she complained of numbness on both sides. Her articulation was indistinct; she became more and more insensible, and sank, completely comatose.

No positive disorganization of the brain could be detected. The ventricles, however, contained more than usual serum; and there were found thickening and increased vascularity of the membranes, with moderately firm adhesion in some parts; in others, an apparently gelatinous, transparent and colorless deposit interposed between them.

Unfortunately, no examination of the spinal cord was made.

In this case there was very likely, as in my experiments, an increase in the temperature of the side paralyzed of motion. The writer merely says that *the patient was constantly complaining of an uncomfortable sense of heat.* There was, as in my animals, an evident increase in sensibility on that side.

M. Monod* has related the case of a man who, after having felt a sudden pain in his back, became paralyzed in the motion of the right inferior limb. Sensibility was entire on this side, but on the left side, where the movements were entire, sensibility was entirely lost from the breast to the foot. There was at first no fever. The patient died 34 days after the beginning of this affection.

The brain and its membranes were normal. A hemorrhage

* Bulletin de la Société Anatomique, No. xviii. p. 349.

had taken place, and blood was found in the right side of the central gray matter, in the neighborhood of the anterior column in the dorsal and lumbar regions.

This case is assuredly a very remarkable one, and in accordance with my experiments.

The conclusion to be drawn from these four cases is, that in man as well as in animals, there appears to be a crossing of the sensitive nerve-fibres in the spinal cord.

### XXX.—ON THE DIFFERENT DEGREES OF EXCITABILITY OF THE DIFFERENT PARTS OF THE SENSITIVE NERVE-FIBRES.

It is a well-known fact, that an excitation of the skin or of a mucous membrane, produces a greater pain or a greater reflex action than that of the nerve trunk, from which these parts receive their nerve-fibres. For instance, a slight excitation of the laryngeal mucous membrane produces coughing, while an excitation of the vagus nerve very rarely produces the same effect. Therefore, there is a notable difference between the peripheric extremity of a nerve-tube and its part contained in a nerve-trunk.

The existence of a peculiar organ in the skin (the *corpuscles of touch* of Wagner) has not much (if it has anything) to do with the different degrees of excitability of nerve-tubes in the skin and in the trunks of nerves. The corpuscles of touch do not exist in the mucous membranes, and if they exist in the skin of frogs, turtles, etc., it is in a very small number ; and, nevertheless, the degree of excitability of nerve-fibres in these parts is much superior to that of the fibres of the nerve-trunks.

Some very striking differences exist in the degree of excitability of centripetal nerve-fibres in the five following different parts of their length.

1st. The part contained in the skin.

2d. The part of a nerve extending from the skin to the spinal cord.

3d. The posterior roots of the spinal nerves.

4th. The part of the posterior roots attached to the spinal cord.

5th. The part of the cutaneous nerve-fibres contained in the gray matter of the spinal cord.

The fibres existing in the gray matter of the spinal cord appear to be inexcitable, at least by our ordinary means of excitation. Of the four other parts, the less excitable is the nerve between the ganglion and the skin. The excitability of the posterior roots is less than that of the skin and that of their part attached to the spinal cord. Of these two last parts the skin is less excitable than the other.

I measured the excitability by the degree of pain or of reflex action. The differences are much more easily found for the reflex action than for the pain.

Is it because they have been connected with the cells of the central gray matter of the spinal cord, that the centripetal fibres, contained in that gray matter, are not excitable? If it is so, there is a difference between these cells and those of the ganglions on the posterior roots, because the connection of these fibres with the cells of these ganglions does not prevent their being excitable.*

From the facts above related I conclude that the same nerve-fibre, in different parts of its length, may have very different degrees of excitability.

(To be continued.)

* Recently, Dr. Cl. Bernard has communicated to the *Société de Biologie*, of Paris, a fact which would be very important if it were exact. He says that some of the fibres of the posterior roots of the spinal nerve, in frogs, do not pass through the ganglion,—that they are the sensitive fibres, and that the ganglionic fibres are merely for reflex action. I have made, alone or with my friend Dr. Henry Lolliot, the minutest examination of the spinal ganglions, and I am satisfied that all the fibres of the posterior roots pass in the ganglion, and that Dr. Bernard has been mistaken.

